# Listeners Exploit Syntactic Structure On-Line to Restrict Their Lexical Search to a Subclass of Verbs

**DOI:** 10.3389/fpsyg.2015.01841

**Published:** 2015-12-15

**Authors:** Perrine Brusini, Mélanie Brun, Isabelle Brunet, Anne Christophe

**Affiliations:** ^1^Language, Cognition and Development Lab, Cognitive Neuroscience Department, Scuola Internazionale Superiore di Studi AvanzatiTrieste, Italy; ^2^Laboratoire de Sciences Cognitives et de Psycholinguistique, École des Hautes Études en Sciences Sociales (EHESS), Centre National de la Recherche Scientifique, École Normale Supérieure (ENS)Paris, France; ^3^Laboratoire Psychologie de la Perception, Université Paris DescartesParis, France; ^4^Département d'Etudes Cognitives, Ecole Normale Supérieure - PSL Research UniversityParis, France

**Keywords:** linguistic expectation, verb argument structure, lexical search, on-line syntactic structure construction

## Abstract

Many experiments have shown that listeners actively build expectations about up-coming words, rather than simply waiting for information to accumulate. The online construction of a syntactic structure is one of the cues that listeners may use to construct strong expectations about the possible words they will be exposed to. For example, speakers of verb-final languages use pre-verbal arguments to predict on-line the kind of arguments that are likely to occur next (e.g., Kamide, 2008, for a review). Although in SVO languages information about a verb's arguments typically follows the verb, some languages use pre-verbal object pronouns, potentially allowing listeners to build on-line expectations about the nature of the upcoming verb. For instance, if a pre-verbal direct object pronoun is heard, then the following verb has to be able to enter a transitive structure, thus excluding intransitive verbs. To test this, we used French, in which object pronouns have to appear pre-verbally, to investigate whether listeners use this cue to predict the occurrence of a transitive verb. In a word detection task, we measured the number of false alarms to sentences that contained a transitive verb whose first syllable was homophonous to the target monosyllabic verb (e.g., target “dort” /dɔʁ/ *to sleep* and false alarm verb “dorlote” /dɔʁlɔt/ *to cuddle*). The crucial comparison involved two sentence types, one without a pre-verbal object clitic, for which an intransitive verb was temporarily a plausible option (e.g., “Il dorlote” / He *cuddles*) and the other with a pre-verbal object clitic, that made the appearance of an intransitive verb impossible (“Il le dorlote” / *He cuddles it*). Results showed a lower rate of false alarms for sentences with a pre-verbal object pronoun (3%) compared to locally ambiguous sentences (about 20%). Participants rapidly incorporate information about a verb's argument structure to constrain lexical access to verbs that match the expected subcategorization frame.

## Introduction

To understand spoken sentences, listeners have to process speech sounds, recognize words and morphemes, and decode the syntactic structure of the sentence to recover its meaning. All of these complicated processes seem effortless and are performed in a very short amount of time (Pylkkänen and Marantz, 2003; Poeppel et al., 2008). One way to explain the speed with which spoken language is processed is to suppose that the human language parser is able to exploit the context—both linguistic and non-linguistic—to compute expectations about upcoming material, thus reducing the number of possible options available at any given point in time and anticipating the processes it is likely to have to complete next (e.g., Levy, 2008; Gibson et al., 2013).

Many experiments have shown that listeners indeed use context to build linguistic expectations (see e.g., Kamide, 2008; Rayner, 2009, for reviews). The use of lexico-semantic knowledge to anticipate upcoming words was one of the first studied phenomenon: comprehenders were shown to use the beginning of a sentence to look faster to likely referents (e.g., Altmann and Kamide, 1999), to read predictable words faster (e.g., Frisson et al., 2005), and they also displayed smaller N400 responses to content words that were made more likely by their preceding contexts (e.g., Kutas and Hillyard, 1980; Federmeier, 2007; Xiang and Kuperberg, 2015). Once they've generated a prediction about a possible upcoming content word, listeners can also build specific expectations regarding the phonological shape of the article that precedes it (DeLong et al., 2005), or the gender of a preceding adjective or article (Wicha et al., 2004; Van Berkum et al., 2005; Foucart et al., 2015). In addition, they are able to integrate other kinds of information within their anticipatory processes, such as the prosodic/rhythmic pattern of a sentence (Brown et al., 2011) or the phonological patterns typical for specific syntactic categories (Farmer et al., 2006).

Syntactic structure *per se* should be another good candidate for activating anticipatory linguistic processes: Indeed many studies have observed that participants are able to extrapolate syntactic information on the fly to build expectations regarding an up-coming word or structure (e.g., Boland et al., 1990; Konieczny, 2000; Kamide et al., 2003; Boland, 2005; Hare et al., 2009; Levy and Keller, 2013), and modeling work shows that probabilistic parsers trained on linguistic corpora account for a whole range of human experimental data (e.g., Jurafsky, 1996; Hale, 2001; Levy, 2008). For instance, comprehenders exploit a verb's argument structure to expect specific kinds of arguments (e.g., Boland et al., 1990), and use the selectional constraints imposed by the verb to predict probable referents (i.e., listeners expect an eatable entity after hearing the verb “to eat,” Altmann and Kamide, 1999). In a verb-final language such as Japanese, a verb's argument structure can be exploited even before the verb is heard, so that the presence of an indirect object, for instance a DP marked with a goal marker, leads the parser to search for a patient before hearing the corresponding DP (Kamide et al., 2003).

This ability to build an argument structure before hearing its head (the verb), might be a specific adaptation from head-final languages comprehenders, to cope with the fact that verbs systematically appear after their arguments, and avoid lengthy delays. It could also, however follow from a general property of the human parser, which would use all the information available to constrain its ongoing syntactic structure on-line. A very recent paper by Omaki and colleagues addressed this precise issue (Omaki et al., 2015). They exploited filler-gap dependency completion in object relative clauses, as in “the city that the author chatted regularly about was named after an explorer,” and measured participants' surprise upon hearing the intransitive verb “chatted” which cannot take a direct object (the preposition “about” provides a slot as an indirect object for “the city” and makes the sentence grammatical in the end). If participants wait until they process the verb in order to posit an object position for that verb, then they should show no surprise upon hearing an intransitive verb (since they haven't attempted yet to assign “the city” to a direct object position), and should simply wait longer until they find a suitable slot for the DP “the city.” Instead, in three experiments, Omaki et al. observed delayed reading (interpreted as delayed processing) upon encountering an intransitive verb in such a position. These results thus strongly suggest that in English too, a verb-medial language, comprehenders posit an argument structure even before they have processed the verb.

In this paper, we will address this same question through a different angle. Rather than looking for the effect of specific content words on the predictability of upcoming words (integrating them within the on-going syntactic structure), we focus on purely structural effects: namely, we wonder whether participants are able to exploit the syntactic structure they've heard so far—irrespective of the semantic content of the specific lexical items involved—in order to build expectations as to the type of word that is likely to occur next. Previous work on this topic has yielded somewhat mixed results: within the noun phrase, Dahan et al. (2000) have shown that after hearing a gender-marked article, listeners successfully reduce their lexical search to gender-matching referents. When ambiguous words of different syntactic categories are involved (e.g., noun/verb, or adjective/noun), some studies have found that both meanings of a homophone are initially activated (Tanenhaus et al., 1979) while others found that context allowed listeners to completely ignore the unintended meaning, when one member of the homophone pair was a function word (Shillcock and Bard, 1993, e.g., “would” vs. “wood”), when the preceding linguistic context marked syntactic constituent boundaries through phrasal prosody (Millotte et al., 2008; de Carvalho et al., 2015), and when the visual context led listeners to expect either an adjective or a noun, for pragmatic reasons (Magnuson et al., 2008). Here, we focus on the level of the verb phrase, and wonder whether listeners are able to use elements from the subcategorization frame of a verb in order to constrain lexical access to upcoming verbs.

To do so, we tested whether the presence of a preverbal direct object pronoun blocks the activation of intransitive verb candidates, or not. In French, as in other Romance languages, the direct object of a verb is pronominalized as a clitic accusative pronoun (“le,” “la,” or “les”) that rises in the left periphery of the transitive verb (Kayne, 1991). For example the DP complement of the French verb “manger” *to eat*, “la souris” in “Le chat mange la souris” *The cat eats the mouse* moves to the left-periphery of the verb when pronominalized, as in “Le chat **la** mange” *the cat eats* ***it***. This situation resembles the one in head-final languages such as Japanese; however, whereas all direct objects are preverbal in Japanese, whether they are pronominalized or not, in French only pronoun direct objects are pre-verbal, while full DP objects appear post-verbally. Thus, in French the presence of pre-verbal objects is not a standard configuration (although it is reasonably frequent). Consequently, if French listeners are able to integrate the object pronoun clitic into the syntactic structure, and deduce on-line that this utterance calls for a transitive verb, this will confirm Omaki et al.'s finding that the fast integration of pre-verbal arguments is a general ability of the human parser, rather than a specific adaptation from listeners of head-final languages where objects systematically appear pre-verbally. Additionally, such a result would enlarge the growing body of evidence that the human parser is processing a wide variety of available cues in order to anticipate the linguistic material that might follow. In contrast, if French listeners do not make use of pre-verbal object pronouns to anticipate transitive verbs, this will suggest that the ability to anticipate upcoming materials is fine-tuned to the specific properties of the language being processed, such that only features that are usually relevant will be put to use by the parser.

We investigated this question using a false alarm paradigm with adult French speakers. Subjects were instructed to respond as quickly and accurately as possible to an intransitive monosyllabic verb (e.g., “dormir” *to sleep*, that surfaces as “il dort” /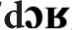/ *he sleeps* when conjugated in the 3rd person singular present tense). While half the sentences did contain the target verb, the other half did not contain it, but contained instead a multisyllabic transitive verb that started with the same first syllable (e.g., “dorloter” *to cuddle*, that surfaces as “il dorlote” /
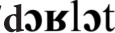
/ when conjugated in the 3rd person singular present tense). The number of false alarms triggered by this multisyllabic verb was the measure of interest here. To test the impact of the under-construction syntactic structure upon lexical access, we inserted this multisyllabic catch verb in two kinds of sentences: In the first experimental condition, it appeared immediately after the pronoun subject, and was followed by an object DP (e.g., “elle dorlote son nounours” *she cuddles her teddybear*); thus, at the point when the verb was processed, the available information was still compatible with an intransitive verb (only the pronoun subject had been heard), and the intransitive target verb was thus a plausible option to continue this sentence (e.g., “elle dort toute la nuit” *she sleeps through the night*). We expected this condition to trigger a baseline amount of false alarms. In the second condition, the catch verb appeared after a pronoun subject and an object clitic (e.g., “elle le dorlote toute la nuit” *She cuddles it through the night*). In that case, when listeners heard the beginning of the verb, they had already heard the clitic object: if they spontaneously integrate this clitic object on-line to the syntactic structure they are building, they should be able to reject the intransitive target verb as a possible continuation for that sentence, and should therefore exhibit a very low proportion of false alarms, close to zero. If, in contrast, lexical access is primarily based on the available phonological information (here, the first syllable of the catch verb, which matches the target verb), together perhaps with a coarse syntactic information (e.g., that a verb is expected), then the proportion of false alarms triggered should be roughly equal in both conditions, irrespective of the fact that the second one contains a pre-verbal object clitic and the first one does not.

## Materials and methods

### Participants

Twenty-five native speakers of French took part in this experiment and were paid 5€ for their participation. Two additional participants were tested but their data were discarded from the final analysis because their hit rate was too low (<30%). This work was approved by the local ethics committee (Paris Ile de France III), and all participants signed an informed consent form.

### Stimuli

Using the LEXIQUE 3.55 database (New et al., 2001), we selected 14 pairs of verbs consisting of an intransitive monosyllabic verb (or, more precisely, a verb that could not take a direct object), and a multisyllabic transitive verb whose first syllable was homophonous to the intransitive monosyllabic verb. For example, the verb “dormir” *to sleep* was paired with the verb “dorloter” *to cuddle*. While both “il dorlote…” *he cuddles…* and “il le dorlote” *he cuddles it* are grammatical structures, the sequence “^*^il le dort” *he sleeps it* is ungrammatical (see Supplementary Material for a complete list of the verbs). Only the intransitive verb of each pair was used as a target in the word detection task.

Each pair of verbs was used to build one or several quadruplets of sentences, for a total of 31 quadruplets. Each quadruplet contained two HIT sentences that actually contained the target monosyllabic intransitive verbs (e.g., “Quand il fait nuit, elle dort tranquillement,” *During the night, she sleeps peacefully* and “Quand il fait nuit, elle dort dans son lit,” *During the night, she sleeps in her bed*), as well as two False Alarm sentences that contained the multisyllabic transitive verb. One of these false alarm sentences contained a pre-verbal object pronoun (“le,” “la,” or “les” *it masc, fem, plural*): this created an ungrammatical context for an intransitive verb (as in “Quand il fait nuit, elle **la** dorlote plus,” *During the night, she cuddles it more*). The other false alarm sentence was locally ambiguous, in that the verb immediately followed a subject DP (which could be a personal pronoun), such that both members of a verb pair, the transitive and the intransitive one, were compatible with the structure heard so far (e.g., “Quand il fait nuit, elle dorlote sa poupée,” *During the night, she cuddles her doll*). Crucially, all sentences from a quadruplet contained the same sequence of words before the verb phrase (“Quand il fait nuit, elle…” in the examples): this ensures that the only pre-verbal cue that can be used to constrain lexical access to the verb is the object clitic (when it is present). In other words, if the false alarm rate is greater for locally ambiguous sentences than for non-ambiguous sentences (with an object clitic), this can only be due to the fast integration of the pronoun object clitic which makes subjects discard the possibility of encountering an intransitive verb.

To check whether or not there were acoustic/prosodic differences across conditions on the syllable homophonous with the target word, we measured the duration and F0 of the first syllable of the multisyllabic verbs of the false alarm sentences (e.g., “dor” from “dorlote”). We compared these values with a Wilcoxon rank test (since visual inspection showed that the duration and F0 values were not normally distributed).The results of this analysis are presented in Table 1. We observed a marginally significant effect of duration, with FA_CLI sentences tending to have somewhat shorter first syllables than FA_AMB sentences (about 10 ms). It is unlikely that such a small difference would trigger a major difference in False Alarm rates between the two conditions.

**Table 1:** **Mean and standard error for the duration and F0 of the first syllable of the multisyllabic carrier verb from the false alarm sentences (e.g., “dor” in “dorlote”)**.

	**FA_CLI mean (std. error)**	**FA_AMB mean (std. error)**	**Wilcoxon rank test *Z*(*p*)**
Duration (ms)	173 (9.63)	183.5 (9.79)	−1.86 (*p* = 0.062)
Mean F0 (vowel) (Hz)	245.4 (4.89)	255.8 (4.16)	−0.975 (*p* > 0.3)

The 31 quadruplets thus amounted to a total of 124 test sentences. Experimental sentences were recorded by an expert speaker (the last author) and marked at the onset of the critical verb using PRAAT (Boersma and Weenink, 2015). Each participant heard each of the 124 experimental sentences once within two blocks of 62 sentences: for each quadruplet, one block contained one HIT and one ambiguous False Alarm sentence (FA_AMB), while the other block contained the other HIT sentence and the non-ambiguous False Alarm sentence, which featured a pre-verbal clitic (FA_CLI). Each block contained roughly the same amount of FA_AMB and FA_CLI sentences. In total, a subject was exposed to 62 HIT sentences, 31 FA_CLI and 31 FA_AMB sentences. Within each block, the order of the sentences was pseudo-randomized so as to avoid sequences of five or more false alarm sentences.

### Procedure

Each participant was tested individually. They were instructed to press the spacebar from a computer keyboard as soon as they could identify the target verb. A trial began with the visual presentation of the target word, always an intransitive verb written in the infinitive form (1.5 s), followed by a black screen with a white fixation cross (1 s), then a sentence was played (the auditory stimuli were stored at a sampling rate of 22,050 Hz and were presented through headphones). The trial ended 2.5 s after the subject's response or after the end of the auditory presentation (whichever came first), and a new trial began immediately. Response times were measured from the onset of the target word. Speed and accuracy were emphasized in the instructions. Before the experiment began, participants performed a short training. If they gave an incorrect or delayed response during training (more than 1 s response time), a warning message appeared on the screen asking them to correct or speed up their response (depending on the situation). The whole experiment was run using the Psychotoolbox of Matlab (Kleiner et al., 2007) and lasted about 15 min including a pause (of about 2 min) between blocks.

### Analysis

Since the false alarm responses were categorical (0 for no response, 1 for a FA), we used a logit model to analyze whether false alarms were distributed differently between the FA_CLI and FA_AMB conditions. We ran a mixed model analysis using R 3.2 and the lme4 package (v 1.1-6, based on Bates and Sarkar, 2007). Each false alarm *F*_*isc*_, for a given item i (where an item represents the pair of False Alarm sentences from the same quadruplet, i varied between 1 and 31) and a given subject s (between 1 and 25), in a given Condition c (FA_CLI vs. FA_AMB), was modeled via an intercept β_0_ reflecting the baseline probability of making a false alarm, and a slope estimate β_1_ of the predictor variable C (Condition), reflecting the impact of the context on the probability of making a false alarm (either a locally ambiguous context, FA_AMB condition, or non-ambiguous context with a pre-verbal clitic that makes an intransitive verb unlikely, FA_CLI condition). Since we used the maximal random effect structure (recommended by Barr et al., 2013), we also included by-subjects and by-items intercepts (S_0s_ and I_0i_ allowing the baseline to vary from β_0_ by a fixed amount for each subject s and each item i) and slopes (S_1s_ and I_1i,_ respectively, allowing each subject and item to deviate from the population slope β_1_in their sensitivity to Condition). The categorical predictor Condition C was coded as 0 for the ambiguous context (FA_AMB) and 1 for the object pronoun context (FA_CLI). The resulting equation for the model, taking into account a normally distributed error for each observation, e_*is*_, is the following:

(1) $$\begin{array}{l} \text{Logit}\left(\text{P}\left(F_{is}=1\right)\right)=\beta_{0}+S_{0\text{s}}+I_{0\text{i}}+\left(\beta_{1}+S_{1\text{s}}+I_{1\text{i}}\right).\;C+e_{\text{is}} \end{array}$$

β estimates are given in log-odds (the space in which the logit models are fitted). To compare the probabilities of making a false alarm across the two levels of C (Conditions: ambiguous context vs. non-ambiguous object clitic pronoun context), we computed the difference: P(*F*_*is*_ = 1; *C* = 0 i.e., FA_AMB) – P(*F*_*is*_ = 1; *C* = 1 i.e., FA_CLI) by taking the inverse logit of the right-hand side of Equation (1).

We computed Wald's Z statistic using the mixed model described above. This statistic tests whether the estimates are significantly different from 0. Hence the intercept corresponds to the probability of making a false alarm when participants are exposed to an ambiguous context, while the slope corresponds to the modification in the probability of making a false alarm when participants hear an object clitic before the verb.

## Results

**Figure 1:**
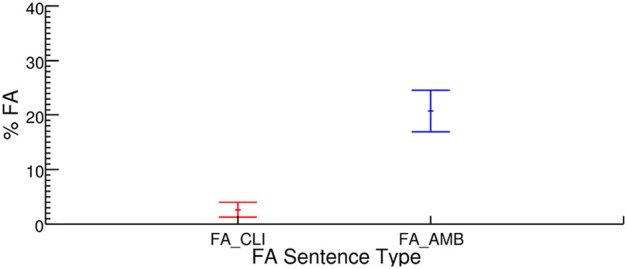
**Percentage of false alarms made by participants for each type of false alarm sentence presented, non-ambiguous FA sentences with a clitic (FA_CLI) in red (left-hand side) and ambiguous FA sentences (FA_AMB) in blue (right-hand side)**. Error bars represent the standard error of the mean (by participants).

The Hit rate was 90.8%, with an overall False Alarm rate of 11.6% (averaged across FA_CLI and FA_AMB conditions), showing that participants performed the task adequately. To assess whether or not French listeners quickly integrate the presence of a clitic object pronoun in order to compute the probability of occurrence of a transitive vs. intransitive target verb, we compared the false alarms produced by subjects when they were exposed to non-ambiguous FA_CLI sentences containing a clitic object (as in “Quand il fait nuit, elle la dorlote plus” *During the night, she cuddles it more*) to ambiguous FA_AMB sentences that did not contain a clitic object (as in “Quand il fait nuit, elle dorlote sa poupée,” *During the night, she cuddles her doll*). The mean proportion of false alarm responses is plotted in Figure 1. As can be seen, subjects made many more false alarms to ambiguous sentences, presenting a syntactic context that is appropriate for both the transitive and intransitive verbs (20% false alarms, range 3.23–41.94%, by participants), than to non-ambiguous sentences featuring a clitic object pronoun: sentences of this type only triggered 3% of false alarms (range: 0–9.68%). This result was confirmed by our mixed model analysis exhibiting a main effect of the predictor Condition (β = −3.01; *z* = −4.35; *p* = 1.4e-05) corresponding to a decrease of 0.16 in the probability to make a false alarm when the participant heard an object pronoun clitic (FA_CLI condition) relative to when there was no object pronoun (FA_AMB condition).

Thus, the probability that participants would be influenced by the sound similarity between the target verb (intransitive) and the verb that was actually present in the sentence (multisyllabic transitive), was largely reduced by the presence of the pre-verbal object clitic. As pointed out by a reviewer, every sentence which exhibited a pre-verbal clitic object did not contain the target. As a result, one may wonder whether the reduced rate of false alarms on FA_CLI sentences with a pre-verbal object did not result from participants learning, over the course of the experiments, that clitic objects signaled sentences without a target. If this were the case, the difference in False Alarm rates between conditions should start at zero and increase with time, as participants start using the strategy. To examine this possibility, we checked whether the difference in false alarm rates increased between the two experimental conditions, over the course of the experiment. Table 2 shows the percentage of False Alarms, in both conditions, for each quarter of the experiment (first 31 trials, trials 32–62, trials 63–93, and 94–124).

**Table 2:** **Percentage of False Alarms, in both conditions (FA_AMB and FA_CLI), for the 4 quarters of the experiment**.

	**FA_AMB (%)**	**FA_CLI (%)**
1st quarter	32	7.5
2nd quarter	23	2
3rd quarter	15	0.9
4th quarter	18	0.3

As can be seen, the difference in proportion of False Alarms between conditions does not increase with time. The only observable effect is a sharp decrease in overall False Alarm rate, as the experiment unfolds, suggesting that as participants became aware that they got caught on some of the false alarm sentences, they adopted a more conservative response bias. However, this pattern occurs for both types of False Alarms. In particular, there is already a massive difference between the FA_AMB and FA_CLI conditions in the first quarter of the experiment, suggesting that the lower rate of responses in the FA_CLI condition is present from the start, and therefore unlikely to be the result of a specific strategy developed as a function of the experiment (specifically, noticing that whenever a clitic is present the target is not there).

All in all, these results show that subjects dismiss the possibility that the target intransitive verb will occur, when they process the clitic object pronoun, an argument that is incompatible with the target intransitive verb.

## Discussion

We investigated the ability of French listeners to quickly compute the match or mismatch between the subcategory of an upcoming verb, and the presence or absence of a pre-verbal direct object pronoun. The logic here is that if French listeners are able to rapidly integrate the information conveyed by the object pronoun, they should be able to completely rule out an intransitive verb as a possible continuation of that sentence—since, by definition, an intransitive verb cannot take a direct object. We observed that participants' tendency to falsely detect a monosyllabic intransitive target verb was much higher when the multisyllabic carrier verb occurred in a syntactic context which was congruent with the target (e.g., “elle dorlote…” *she cuddles…*, FA_AMB condition), than when the carrier verb was preceded by an object pronoun (e.g., “elle le dorlote”/ *she cuddles it*, FA_CLI condition), making the syntactic context impossible for the target verb (“^*^elle le dort”/^*^she sleeps it). This result shows that the participants integrated the clitic object pronoun on-line into the syntactic structure of the sentence, and inferred from this that the target intransitive verb would not follow.

This study confirms, with a different experimental technique, a different language, and in a different modality (listening vs. reading), the results obtained by Omaki et al. (2015) in English: They studied filler-gap dependency completion in object relative clauses and observed that processing of “chatted” was slowed down in a sentence such as “the city that the author chatted regularly about was named after an explorer,” because there was a mismatch between the verb “chatted” which cannot take a direct object, and the implicit assumption that “the city” will be the direct object of the next encountered verb. Omaki et al. concluded from their series of three experiments that English-speaking comprehenders build argument structure before having heard the verb itself—just like Japanese-speaking comprehenders do, even though English is a verb-medial language. In their discussion, however they acknowledge the fact that their data are compatible with an alternative interpretation in which argument structure building does not occur ahead of the verb (p. 14). Under that alternative interpretation, participants would initially access only very coarse category information about the verb, namely that it is a verb; at that first step, filler retrieval processes would be activated and an object filler would be posited for the verb; only later would finer-grained information about the verb subcategory be retrieved, transitivity information would then become available and reveal the mismatch between the filler and the verb. In our experimental design, this alternative processing strategy would have led to opposite effects: As we mentioned in our introduction, if participants initially generated expectations about lexical items on the basis of coarse category information (e.g., Verb, Noun), then the two contexts, with and without an object clitic, should have led to approximately the same number of false alarms. Indeed, both are equally good verb contexts, and should have led participants to occasionally respond too fast upon hearing a word starting with the target verb, in a verb position. The fact that participants made almost zero false alarms to the sentences with an object clitic shows that they were able to compute that this context was inappropriate for the target intransitive verb, even before they had started hearing the first phonemes of the verb itself. Taken together, the available experimental evidence thus suggests that comprehenders' ability to exploit pre-verbal arguments to constrain their interpretation of sentences, even before they have heard the verb itself, is not a specific adaptation to verb-final languages, but reflects instead a more general behavior of the human language parser.

Note that two mechanisms are compatible with the present set of results: either a predictive account, in which the preceding context is used to generate specific expectations about upcoming words, which are then matched with the input; or an integrative account, in which the preceding context is integrated very rapidly with the available phonological information. As we mentioned above, the fact that almost zero False Alarms were observed in the FA_CLI condition suggests that participants were able to compute that the target was unlikely to occur even before they heard its first syllable, which might be interpreted as evidence in favor of the predictive account over the integrative account. However, since the target verb was specified as a target before the sentence itself, it is likely that it was pre-activated before participants even started to process the sentence. As a result, the integration between context and target verb could start even before the first syllable of the carrier verb was heard, because the target verb was pre-activated. In other words, at every point in time, participants could try to work out whether their target word is likely or not in that context. When it is consistent with the context (as in FA_AMB sentences), it is likely to occur next, and hearing consistent phonological information probably results in the increased rate of False Alarms. When it is not consistent with the context (as in FA_CLI sentences), then it is unlikely to occur, and the processing of phonological information in the hope of finding the target verb may stop very early (and result in the almost-zero rate of false alarms we observed). In other words, the task we used makes it possible for participants to integrate the preceding context both with the phonological information as it becomes available, and the information about the target verb that was provided before the sentence began. All in all, both the predictive and the integrative interpretations account equally well for the present set of results^1^.

An interesting particularity of the experimental paradigm we chose to use is that it allowed us to test participants' ability to use abstract syntactic information, in the absence of any semantic information conveyed by content words. Indeed, in the sentence quadruplets that were used, the first words of all four sentences, up to the critical word, were always identical, and the only difference was the presence or absence of the clitic object pronoun just before the critical verb. Because there was no difference whatsoever in the content words that were heard before the critical verb, participants' behavior was thus necessarily due to their processing of the syntactic role of the object pronoun. Thus, listeners can exploit the syntactic structure they are constructing on-line to restrict their lexical search to word candidates that fit this syntactic structure.

This conclusion might seem at odds with recent results from Chow et al. (2015), in which they conclude that comprehenders initially rely on the lexical meanings of arguments—but not their structural roles—to compute predictions about a likely upcoming verb (using sentences in which arguments were reversed, e.g., “which customer the waitress had served,” vs. “which waitress the customer had served”). In the present experiment, we conclude that listeners exploit the structural role of an argument—e.g., direct object—to infer whether a target intransitive verb is plausible or not in that context. The apparent discrepancy here comes from the difference in experimental paradigms, which tested different kinds of inferences about the upcoming verb. In Chow et al. (2015) what was delayed was not really the computation of a structural role of an argument (e.g., subject, or direct object), but rather the computation of an argument's likely thematic role based on its structural role. For instance, if an argument occupies the subject position, is it the agent of the action or not? Often yes, but not necessarily (depending on the nature of the verb and the structure of the sentence). In the present experiment, simply knowing whether the verb takes a direct object or not (irrespective of the thematic role played by the referent occupying that position), was sufficient to constrain lexical access and eliminate the intransitive candidate. In Chow et al. (2015) participants had not only to find events involving waitresses (which might be fast), but also to find events in memory involving waitresses as agents (which might be slower). So overall, the available evidence suggests that structural roles are computed fast, and exploited on-line, so long as this does not involve an extra step (assigning thematic roles to arguments, and/or retrieving specific event types in memory).

If all the experiments presented above clearly point out the capacity of the linguistic parser to exploit various features of its input to anticipate different aspects of upcoming materials, it remains unclear to what extent these phenomena actually occur outside of the lab. Indeed, within experiments, participants are often placed in closed-choice situations, which restrict the number of possible anticipations that might be entertained. For the experiment reported here, participants are presented with the target verb before hearing a sentence, thus reducing considerably the possible verbs that are expected. The same issue can be addressed to the “visual-word” paradigm, where participants are exposed to a finite number of images representing the sentence they are exposed to. As an aside, this is not such an artificial situation, as people in real-life situations will often have access to other elements of context, either visually (with a less impoverished visual context than in the visual-world paradigm), or through preceding linguistic materials (with a small set of words that can be made highly plausible by the preceding context). Even in reading experiments in which no visual or discourse context is available, participants are repeatedly exposed to sentences with similar syntactic structures, which may restrict the kind of materials they are led to expect—and participants do exploit this kind of experiment-specific information (see e.g., Gibson et al., 2013). However, all these results clearly show the capacity of the parser to rapidly integrate useful information to facilitate the process of on-line comprehension. Future research should investigate how this type of anticipation processes can be used in less constrained situations.

To conclude, the study reported here focused on the ability of French listeners to rapidly integrate syntactic cues to constrain lexical access and eliminate verb candidates on the basis of a mismatch between their sub-category and the syntactic context. Participants were shown to be able to take into account a very subtle cue, a clitic object pronoun, to infer that a target intransitive verb was unlikely to come next. This result proves that the human language parser can use subtle syntactic cues to constrain lexical access on-line, and restrict the lexical search to candidates that fit the ongoing syntactic structure. This study nicely aligns with previous data suggesting that each element from the input can be analyzed and exploited by listeners, on-line, to improve the precision of linguistic processing at all levels of linguistic analysis.

### Conflict of interest statement

The authors declare that the research was conducted in the absence of any commercial or financial relationships that could be construed as a potential conflict of interest.

## Footnotes

^1^We thank a reviewer for helping us to clarify this important point.
