# Supplementary material for: Listeners Exploit Syntactic Structure On-Line to Restrict Their Lexical Search to a Subclass of Verbs
Source: Front Psychol. 2015 Dec 15;6:1841. doi: 10.3389/fpsyg.2015.01841 (PMC4678230; doi:10.3389/fpsyg.2015.01841)
Supplement: Supplementary file 1 [file DataSheet1.DOCX]

***Supplementary Material***

**Listeners exploit syntactic structure on-line to restrict their lexical search to a subclass of verbs.**

Perrine Brusini^1,^^3*^, Mélanie Brun^3,4^, Isabelle Brunet^2,3^ & Anne Christophe^2,3^

^1^Language, Cognition and Development Lab, Cognitive Neuroscience department, Scuola Internazionale Superiore di Studi Avanzati - via Bonomea, 265 - 34136 Trieste ITALY

^2^ Département d’Etudes Cognitives, Ecole Normale Supérieure - PSL Research University, 29 rue d’Ulm, 75005 Paris, France

^3^ Laboratoire de Sciences Cognitives et de Psycholinguistique (EHESS, CNRS, ENS), 29 rue d’Ulm, 75005 Paris, France

^4^ Laboratoire Psychologie de la Perception, Université Paris Descartes, Paris, 45 rue des Saints-Pères, 75006 Paris, France

*** Correspondence :** Perrine Brusini, office 235, Cognitive Neuroscience department, Scuola Internazionale Superiore di Studi Avanzati - via Bonomea, 265 - 34136 Trieste ITALY

[pbrusini@gmail.com](mailto:pbrusini@gmail.com)

**Annexe**

Sentences from the experiment: a total of 31 quadruplets were constructed from 14 pairs of intransitive/transitive verbs (between 1 and 3 quadruplets per verb, depending on how easily semantically plausible sentences could be constructed).

For each quadruplet, the first two sentences are the false alarm conditions (FA_AMB, locally ambiguous followed by FA_CLI, with a preverbal object clitic and therefore non-ambiguous). The last two sentences are the HIT condition in which the intransitive verb is actually present.

**dormir / dorloter**  -- *to sleep / to cuddle*

1. Quand il fait nuit, elle dorlote sa poupée. *At night, she cuddles her doll.*

Quand il fait nuit, elle la dorlote plus. *At night, she cuddles it more.*

Quand il fait nuit, elle dort tranquillement. *At night, she sleeps peacefully*

Quand il fait nuit, elle dort dans lit. *At night, she sleeps in her bed.*

2. Pour se rassurer, il dorlote son nounours en s'endormant. *To reassure himself, he cuddles his teddybear while falling asleep*

Pour se rassurer, il le dorlote contre son cœur. *To reassure himself, he cuddles it against his heart.*

Pour se rassurer, il dort auprès de son maître. *To reassure himself, he sleeps close to his master (clearly a dog in that instance, in French).*

Pour se rassurer, il dort dans le lit de ses parents. *To reassure himself, he sleeps in the bed of his parents.*

3. Le jeune enfant dorlote le chien du gardien et sa mère ne l’apprécie pas. *The young child cuddles the janitor’s dog and his mother does not appreciate it*.

Le jeune enfant le dorlote tendrement. *The young child cuddles it tenderly*.

Le jeune enfant dort après manger. *The young child sleeps after having eaten*.

Le jeune enfant dort dans le hamac du jardin. *The young child sleeps in the hamac in the garden*

**pleuvoir / pleurer** -- *to rain / to cry*

4. Depuis le début de la semaine, il pleurait son chien fidèle.

Depuis le début de la semaine, il la pleurait dès qu'il était seul.

Depuis le début de la semaine, il pleut mais Pierre doit sortir quand même.

Depuis le début de la semaine, il pleut comme ça a été annoncé à la météo.

5. Il pleurait sa fiancée.

Il la pleurait mais c'est inutile.

Il pleut et cela me rend triste.

Il pleut et les enfants sont mouillés.

**naître / négocier** -- *to be born / to negociate*

6. Martin négocie le prix de sa voiture.

Martin la négocie une nouvelle fois, son augmentation, auprès de son patron.

Martin nait dans une période agitée.

Martin nait dans une famille aisée.

**aller (present tense ‘va’) / vacciner**  -- *to go / to vaccine*

7. Il vaccine toute la population de la ville.

Il le vaccine avant son voyage.

Il va vite manger quand sa mère l’appelle.

Il va rejoindre ses amis au cinéma.

8. Cet après-midi Marie vaccine les enfants de l’école.

Cet après-midi Marie le vaccine contre la grippe.

Cet après-midi Marie va dans le centre pour ses courses.

Cet après-midi Marie va chez le vétérinaire avec son chat.

9. Elle vaccine ses enfants chez le médecin.

Elle la vaccine pendant qu'elle ne regarde pas.

Elle va à l'école par ce sentier.

Elle va au restaurant avec son copain.

**agir / agiter** -- *to act / to wave one’s arms (in that context – can also mean to shake or to wag)*

10. Marie agitait son bras pour appeler le taxi.

Marie les agitait pour qu'on la voie de loin.

Marie agit toujours sur un coup de tête.

Marie agit pour le bien de ses enfants.

**ruer / ruminer**  -- *to kick out / to mull over*

11. Depuis des mois qu'il rumine sa vengeance il va enfin la mettre à exécution.

Depuis des mois qu'il la rumine sa colère, il va finir par être violent.

Depuis des mois qu'il rue devant les obstacles lors de l'entrainement, il ne sera jamais prêt pour la compétition.

Depuis des mois qu'il rue quand les enfants l'approche, il va devoir quitter le poney club.

12. Elle rumine beaucoup sa défaite.

Elle la rumine trop, il faudrait qu’elle parle de sa rancœur à son copain.

Elle rue dès qu'on essaie de la monter.

Elle rue quand le vétérinaire apparaît.

13. Le cerf rumine l'herbe fraiche.

Le cerf la rumine, l'herbe qu'il a mangée au petit matin.

Le cerf rue quand vient la saison des amours.

Le cerf rue quand il sent la présence de chasseurs.

**bouillir / boucher** *-- to boil / to block*

14. Paul bouchait la sortie du garage avec son camion.

Paul le bouchait le tuyau pour éviter une fuite.

Paul bout de l'intérieur.

Paul bout il faut éviter de lui parler.

15. Elle bouchait les trous de ses pantalons avec des écussons.

Elle le bouchait comme elle pouvait.

Elle bout de colère.

Elle bout d'impatience à l'approche des vacances.

**durer / durcir**  -- *to last / to harden*

16. En hiver, le froid durcit la terre et empêche de semer.

En hiver, le froid la durcit et forme de la glace.

En hiver, le froid dure longtemps au Canada.

En hiver, le froid dure toute la journée.

17. Elle durcit le ton pour faire taire les élèves.

Elle le durcit facilement grâce à son nouveau four très puissant.

Elle dure moins longtemps que prévu.

Elle dure pendant deux semaines cette exposition.

**aller (future ira) / irradier** -- *to go / to irradiate*

18. La chaleur irradie toute la pièce.

La chaleur les irradie entièrement.

La chaleur ira au nord demain sous l'influence des courants.

La chaleur ira vers les côtes.

19. Marie irradie les gens de son bonheur.

Marie les irradie pour tester les influences des rayons gamma sur les bactéries.

Marie ira en vacances à la mer.

Marie ira au travail en voiture les jours de grève.

**mentir / manger** -- *to lie / to eat*

20. Il mangeait les tartes de sa grand-mère avec gourmandise.

Il le mangeait tous les jours avec appétit, son gouter.

Il ment à son employeur sur la situation économique.

Il ment souvent sur son âge.

21. Elle mangeait sa glace avec plaisir.

Elle le mangeait avec dégout.

Elle ment sans scrupule au policier.

Elle ment à ses parents, elle a eu une mauvaise note en maths.

22. L'adolescent mangeait beaucoup.

L'adolescent les mangeait voracement ses chips.

L'adolescent ment sur ses fréquentations.

L'adolescent ment c'est lui qui a pris le bijou.

**repartir / reparler** -- *to go back / to speak again*

23. Depuis qu'elle est rentrée chez ses parents, elle reparle politique avec son père.

Depuis qu'elle est rentrée chez ses parents, elle le reparle couramment.

Depuis qu'elle est rentrée chez ses parents, elle repart souvent dans le sud.

Depuis qu'elle est rentrée chez ses parents, elle repart avec eux en vacances.

24. Malgré sa longue absence, il reparle l'espagnol

Malgré sa longue absence, il le reparle avec facilité

Malgré sa longue absence, il repart sans avoir vu sa famille.

Malgré sa longue absence, il repart juste après être arrivé.

**plaire / plaindre (past plaignait)** *-- to please / to pity*

25. Il plaignait ces pauvres gens qui avaient tout perdu.

Il le plaignait d'avoir à le supporter toute la journée.

Il plaît à toutes les filles de son école.

Il plait à sa mère dans ce nouveau costume.

26. Martin plaignait les parents de ces enfants terribles.

Martin le plaignait d'être si mauvais en maths.

Martin plait à sa future belle-mère.

Martin plait à son nouvel employeur.

27. Elle plaignait les vacanciers coincés dans les bouchons.

Elle le plaignait de travailler avec un patron pareil.

Elle plaît beaucoup aux gens.

Elle plaît au serveur, elle a eu une remise.

**muer / mutiler**  -- *to shed its skin (for a snake) / to mutilate*

28. Le serpent mutile sa proie avec ses crochets.

Le serpent la mutile pour la manger.

Le serpent mue pour grandir et atteindre sa taille adulte.

Le serpent mue et abandonne son ancienne peau sans la regarder.

**rire / ridiculiser**  -- *to laugh / to ridicule*

29. À chaque fois, elle ridiculise son secrétaire quand il lui amène son café.

À chaque fois, elle le ridiculise en se moquant de son nez.

À chaque fois, elle rit à gorge déployée.

À chaque fois, elle rit à toutes les blagues même les moins drôles.

30. L'adolescent ridiculise son voisin en montrant du doigt son jean troué.

L'adolescent la ridiculise devant sa bande de copain.

L'adolescent rit en écoutant la radio.

L'adolescent rit beaucoup à la fête foraine.

31. Martin ridiculise sa sœur devant ses amis.

Martin la ridiculise avec ses réflexions désobligeantes.

Martin rit des plaisanteries de son père.

**Martin rit au spectacle de ce fameux humoriste**
